# Multisensory naturalistic decoding with high-density diffuse optical tomography

**DOI:** 10.1117/1.NPh.12.1.015002

**Published:** 2025-01-23

**Authors:** Kalyan Tripathy, Zachary E. Markow, Morgan Fogarty, Mariel L. Schroeder, Alexa M. Svoboda, Adam T. Eggebrecht, Bradley L. Schlaggar, Jason W. Trobaugh, Joseph P. Culver

**Affiliations:** a Washington University in St. Louis, Division of Biological and Biomedical Sciences, St. Louis, Missouri, United States; b Washington University School of Medicine, Mallinckrodt Institute of Radiology, St. Louis, Missouri, United States; c University of Pittsburgh Medical Center, Western Psychiatric Hospital, Pittsburgh, Pennsylvania, United States; d Washington University in St. Louis, Department of Biomedical Engineering, St. Louis, Missouri, United States; e Washington University in St. Louis, Imaging Science Program, St. Louis, Missouri, United States; f Washington University in St. Louis, Department of Electrical and Systems Engineering, St. Louis, Missouri, United States; g Washington University in St. Louis, Department of Physics, St. Louis, Missouri, United States; h Kennedy Krieger Institute, Baltimore, Maryland, United States; i Johns Hopkins University School of Medicine, Department of Neurology, Baltimore, Maryland, United States; j Johns Hopkins University School of Medicine, Departments of Pediatrics, Baltimore, Maryland, United States

**Keywords:** high-density diffuse optical tomography, neuroimaging, functional near-infrared spectroscopy, decoding, naturalistic speech, naturalistic viewing

## Abstract

**Significance:**

Decoding naturalistic content from brain activity has important neuroscience and clinical implications. Information about visual scenes and intelligible speech has been decoded from cortical activity using functional magnetic resonance imaging (fMRI) and electrocorticography, but widespread applications are limited by the logistics of these technologies.

**Aim:**

High-density diffuse optical tomography (HD-DOT) offers image quality approaching that of fMRI but with the silent, open scanning environment afforded by optical methods, thus opening the door to more naturalistic research and applications. Although early visual decoding studies with HD-DOT have been promising, decoding of naturalistic auditory and multisensory stimulus information from HD-DOT data has not been established.

**Approach:**

Audiovisual decoding was investigated using HD-DOT data collected from participants who viewed a library of movie clips. A template-matching strategy was used to decode which movie clip a participant viewed based on their HD-DOT data. Factors affecting decoding performance—including trial duration and number of decoding choices—were systematically evaluated.

**Results:**

Decoding accuracy was 94.2% for four-way decoding utilizing 4 min of data per trial as a starting point. As parameters were made more stringent, decoding performance remained significantly above chance with strong effect sizes down to 15-s trials and up to 32 choices. Comparable decoding accuracies were obtained when cortical sampling was confined to visual and auditory regions and when participants were presented with purely auditory or visual clips.

**Conclusions:**

HD-DOT data sample cortical hemodynamics with sufficient resolution and fidelity to support decoding complex, naturalistic, multisensory stimuli via template matching. These results provide a foundation for future studies on more intricate decoding algorithms to reconstruct diverse features of novel naturalistic stimuli from HD-DOT data.

## 1 Introduction

Decoding of naturalistic information from functional brain imaging data acquired in natural settings has neuroscientific importance for understanding cortical function^1^^–^^3^ and clinical implications for patients with neurological conditions affecting speech and movement—from cerebral palsy to strokes and neurodegenerative disorders.^4^^,^^5^ Studies using recording methods such as functional magnetic resonance imaging (fMRI) and electrocorticography (ECoG) have accurately reconstructed detailed visual and auditory information from cortical brain activity.^3^^,^^4^^,^^6^ For instance, visual cortex activity sampled with fMRI has been used to reconstruct images and movies, providing insight into how these types of information are encoded in the brain and can potentially be extracted to recreate memories, dreams, and real or imagined scenes.^3^^,^^7^^–^^10^ Furthermore, fMRI-recorded responses to narrative stories have been used to map semantic categories across the cortex and accurately reconstruct semantic representations of continuous language.^6^^,^^11^ Meanwhile, intelligible speech has been synthesized from ECoG data,^4^^,^^12^ and accurate and efficient writing has been achieved by decoding intracortical microelectrode recordings,^13^ with promise for patients who are otherwise unable to communicate due to severe speech and motor disorders. However, the widespread application of these findings is limited by the recording methods used. The logistics of MRI scanners are not conducive to purposes such as daily communication, whereas ECoG and intracortical microelectrodes require invasive neurosurgery. To address these limitations, decoding has been investigated using non-invasive and portable technologies such as electroencephalography^14^^–^^16^ and functional near-infrared spectroscopy (fNIRS)^17^^–^^23^ with promising results. However, the traditionally low spatial resolutions of these modalities present a challenge for the nuanced classification of complex naturalistic information. For instance, traditional fNIRS has enabled fascinating decoding research in infants, clinical populations, and brain–computer interfaces but has generally been limited by image quality to coarse classifications between two and six targets.^14^^–^^16^^,^^19^^,^^24^^,^^25^ Applications of naturalistic decoding could benefit greatly from high-resolution brain imaging approaches that can be employed in natural settings.

High-density diffuse optical tomography (HD-DOT) is an emerging high-performance version of fNIRS, which uses high-density optode arrays to provide enhanced image quality that is closer to fMRI than traditional sparse arrays.^26^^–^^29^ Previous work has shown that visual information, such as the location of a checkerboard stimulus, can be reliably decoded with HD-DOT,^30^ capitalizing on accurate retinotopic mapping.^27^^,^^31^^,^^32^ More recently, HD-DOT of the visual cortex has been used to accurately identify silent movie clips using either templates or models.^33^ Furthermore, HD-DOT can map distributed patterns of brain activity in response to audiovisual movies with high image quality across a wide field of view.^34^^,^^35^ However, auditory stimulus decoding and decoding of multisensory stimuli more relevant to real-world contexts have yet to be shown with HD-DOT. Therefore, we aim to evaluate HD-DOT for decoding complex, naturalistic, audiovisual stimuli in this study.

We used a template-matching approach to decode movie clip identity from HD-DOT data in adults viewing a library of audiovisual movie clips. After establishing initial feasibility, we systematically examined how parameters such as the number of templates and trial duration affected decoding performance. To show that multisensory decoding was not driven entirely by either sensory modality in isolation, decoding was also evaluated using data confined separately to either auditory or visual regions of interest (ROIs) and using data from presentations of purely auditory and purely visual versions of the stimuli. This work establishes the feasibility of decoding complex auditory and visual stimulus information from optical neuroimaging data and paves the way for further studies investigating more elaborate decoding with HD-DOT toward goals such as reconstructing natural language and multisensory scenes.

## 2 Methods

### 2.1 Data Set

HD-DOT data were collected from three participants (age 20 to 31 years, female) who viewed up to twenty 5- to 6-min-long animated movie clips twice each over multiple imaging sessions. This resulted in a total of 586.1 min of movie viewing data across three participants, with all participants completing at least 175 min of movie viewing. Participants also completed a series of auditory and visual functional localizers. A subset of these data was reported in a previous publication^35^ and is publicly available on neuroimaging tools & resources colaboratory (NITRC). This previously published data set was expanded to include additional movie viewing runs with consistent data collection and processing methods. Data were ultimately available for decoding analysis from three participants completing at least four imaging sessions that each involved viewing four movie clips twice each. Data collection and preprocessing methods were detailed in the original report, but here, we summarize the aspects most relevant to the current study. Informed consent for participation as well as storage and reuse of data was obtained from all participants in accordance with the IRB protocol approved by the Human Research Protection Office at Washington University School of Medicine.

#### 2.1.1 Imaging system

We imaged participants using an HD-DOT system with 128 laser sources (with wavelengths 685 and 830 nm) and 125 detectors interleaved in a grid with 11 mm spacing, collectively yielding up to 2464 measurements per wavelength within <50-mm source-detector separation, covering posterior, lateral, and dorsal surfaces of the head.^35^ Data were collected at a sampling rate of 10 Hz. The field of view extended ∼1  cm below the pial surface, including regions of the occipital, temporal, parietal, and frontal cortex.

#### 2.1.2 Tasks

The data subjected to decoding analysis were from three adults who watched 5- to 6-min-long audiovisual animated movie clips twice each over the course of multiple imaging sessions. One of the participants completed viewing 20 movie clips twice each over five imaging sessions for a total of 216.9 min of movie viewing. The other two participants completed viewing 16 movie clips twice each over four imaging sessions for a total of 175.6 min of movie viewing for each participant. Each imaging session involved two viewings of each of four unique movies. Supplemental data were collected from one participant who listened to audio clips without visuals and watched silent movies stripped of audio for a control experiment. Auditory and visual functional localizer data from a prior report^35^ were used to generate ROIs. The auditory task involved listening to spoken word lists presented at a rate of one word per second for 15 s followed by 15 s of silence for a total of six blocks per run. For the visual task, participants were asked to maintain central fixation while black and white wedge-shaped checkerboards flickered at 8 Hz in a rotating pattern.^35^ A table detailing the data collected from each participant, as well as the total number of movie viewing minutes, is available in Table S1 in the Supplementary Material.

#### 2.1.3 Data preprocessing

The full details of data preprocessing were as reported previously.^35^ In brief, data cleaning steps included omission of noisy channels with >7.5% variance throughout a run, high-pass filtering (0.02 Hz cutoff) to reduce long-term drift, superficial signal regression to remove superficial tissue signals and global signals including pulse and motion artifacts, and low-pass filtering (0.5 Hz cutoff) to remove residual pulse and high-frequency noise.^26^^,^^35^ Specifically, superficial signal regression involved averaging all first nearest-neighbor measurements, to estimate global scalp signals, and linearly regressing this signal from all measurement pairs. Data were then downsampled to 1 Hz following previous HD-DOT procedures.^26^^,^^30^^,^^35^^,^^36^ Validated HD-DOT data processing streams have traditionally included this downsampling step to streamline the quantity of data being processed as task-related hemodynamics are on the scale of 0.02 to 0.5 Hz.

#### 2.1.4 Data reconstruction

Participant-specific head models, optimized using each participant’s structural MR images and functional localizer HD-DOT data from a previous publication,^35^ were utilized to accurately model light propagation through the head. The light modeling approach employed a finite element, segmented mesh model of the tissue volume and solved the optical diffusion equation over this mesh for the light fluence induced at all tissue locations by the imaging array.^37^^–^^40^ The optical properties at each location were set according to the segmented tissue type there. These tissue types and optical properties are listed in Table S2 in the Supplementary Material and were the same as in a previous study.^35^ This light propagation modeling approach has been extensively validated and provides a more general model than partial pathlength formulations with differential pathlength factors.^39^^–^^43^ The finite element light propagation model yielded Green’s functions and a participant-specific, measurement channels-by-voxels sensitivity matrix A relating the measured light level fluctuations y to the map of changes x in absorption throughout the brain via the linear equation y=Ax.^26^^,^^43^^–^^45^ Each resulting sensitivity matrix was inverted using Tikhonov regularization with $\lambda_{1}=0.05$ and spatially variant regularization with $\lambda_{2}=0.1$ and then used to reconstruct data in a voxelated space. Flat field reconstructions of the sensitivity matrices were thresholded at 10% of their maxima to obtain conservative participant-specific estimates of the field of view, which were later applied as spatial masks during template matching calculations for decoding. Relative changes in oxy- and deoxy-hemoglobin were obtained through spectral decomposition.^26^ Both oxy- and deoxyhemoglobin are used in further analysis, with oxyhemoglobin within the main text and deoxyhemoglobin in the Supplementary Material.

### 2.2 Construction of Auditory and Visual ROIs

To evaluate decoding performance localized to the auditory or visual cortex, we used ROI-confined analyses of decoding performance, employing pre-acquired functional localizer data to define ROIs.^35^ An auditory ROI was constructed by block-averaging word-hearing task data and affine transforming the result to the MNI152 atlas space.^46^ These block-averaged auditory responses were then averaged across all runs and participants, and the map was thresholded at 25% of its maximum value to create a binary mask of the auditory region. Similarly, a visual ROI was established using block-averaged data from the checkerboard-viewing task, and mean responses were thresholded at 25% of their maxima for the left and right hemispheres before being added together for the final binary visual ROI mask. Data were collected, and multisensory decoding was evaluated across the wide field of view of the HD-DOT imaging system to maximize information content for decoding and include cortical regions associated with higher-order processing. Meanwhile, the ROI masks facilitated additional analyses of decoding within cortical regions associated with specific sensory modalities.

### 2.3 Decoding by Template Matching

#### 2.3.1 Timing structure

A simple template-matching approach was adapted from a previous visual decoding study for a first assessment of naturalistic audiovisual stimulus decoding with HD-DOT data.^30^ Each imaging session was analyzed separately to avoid potential errors from cross-subject co-registration. The data from each session were split into two sets—training and testing—with one viewing of each movie clip in each set. Each imaging session contained a minimum of four unique 5- to 6-min movies played twice each. To evaluate the influence that clip duration had on decoding performance, we used a fixed number of templates (four unique movie clips) and systematically increased the trial duration (i.e., the segment of each movie clip utilized for decoding) from 15 to 240 s in increments of 15 s. Alternatively, to evaluate the influence of the number of templates, we fixed the duration of the trials at 45 s and varied the number of templates from 4 to 16 unique templates derived from subdividing the four movie clips. Finally, to further assess the influence that the number of templates and trial duration had on decoding performance, we used different trial durations ranging from 4 min to 15 s, allowing for the number of templates to range from four to 32 for each imaging session. Spatiotemporal templates for each clip were constructed from the training runs, and decoding trials were extracted from the test runs. For both training and testing, temporal feature selection included the removal of the first 15 s of data to avoid transient stimulus onset responses at the beginning of each run.^47^^–^^49^ This temporal cropping also allowed intervening periods between trials to ensure the hemodynamic response from the previous trial had faded. Although the exact timing structure varied between experiments as detailed, the same template-matching approach was used in each case.

#### 2.3.2 Response analysis

The test data were compared with the spatiotemporal (voxels × time) templates for each movie from the training data by calculating the Pearson correlation coefficients $r_{n,m}$ over space and time between the m’th test trial and the n’th template

$$T_{n}^{\prime }=[\begin{array}{c} \begin{array}{c} T_{n}[1,1]-\overset{¯}{\overset{¯}{T_{n}}}\\ \vdots \end{array}\\ T_{n}[v,t]-\overset{¯}{\overset{¯}{T_{n}}}\\ \begin{array}{c} \vdots \\ T_{n}[N_{V},N_{T}]-\overset{¯}{\overset{¯}{T_{n}}} \end{array} \end{array}]\;S_{m}^{\prime }=[\begin{array}{c} \begin{array}{c} S_{m}[1,1]-\overset{¯}{\overset{¯}{S_{m}}}\\ \vdots \end{array}\\ S_{m}[v,t]-\overset{¯}{\overset{¯}{S_{m}}}\\ \begin{array}{c} \vdots \\ S_{m}[N_{V},N_{T}]-\overset{¯}{\overset{¯}{S_{m}}} \end{array} \end{array}],$$ (1)

$$r_{n,m}=\frac{T_{n}^{\prime }\cdot S_{m}^{\prime }}{\left|T_{n}^{\prime }||S_{m}^{\prime }\right|}.$$ (2)

Here, $T_{n}[v,t]$ denotes the amplitude of the n’th template at spatial location (voxel) v and time t within the template, and $S_{m}[v,t]$ similarly denotes the brain response (hemoglobin signal) amplitude in the m’th test trial at voxel v and time t within the trial. $\overset{¯}{\overset{¯}{T_{n}}}$ and $\overset{¯}{\overset{¯}{S_{m}}}$ denote the means of $T_{n}[v,t]$ and $S_{m}[v,t]$, respectively, over all voxels in the region of interest R and all time points in the window of interest W. $N_{V}$ and $N_{T}$ are the number of voxels in R and time points in W, respectively. |a| denotes the Euclidean two-norm of any vector a.

Using a maximum correlation classification approach, the decoding output $D_{m}$ for the m’th trial was finally determined by the template number n that had the maximum correlation with the trial response

$$D_{m}=\underset{n}{\arg\;\max}(r_{n,m}).$$ (3)

Confusion matrices were generated by tracking the number of times that each trial clip was decoded as each of the possible options initially for each participant and imaging session separately. The results were then aggregated by summing the individual confusion matrices across all imaging sessions included in the analysis. Mean decoding accuracy was reported as the total percentage of trials across all sessions that were decoded correctly. Statistical significance was evaluated by comparing the mean decoding accuracy to chance, i.e., 1 / (total number of templates), using a binomial test.^50^ The effect size was evaluated with a Cohen’s d metric: (mean decoding accuracy − chance accuracy) / standard deviation in decoding accuracy over sessions. To evaluate the effects of spatial feature selection using predefined ROI, the template-matching maximum correlation classification method was repeated using the visual-only and auditory-only ROIs. Each ROI was assessed individually by applying the same binary ROI masks to both the training and testing datasets.

**Fig. 1:**
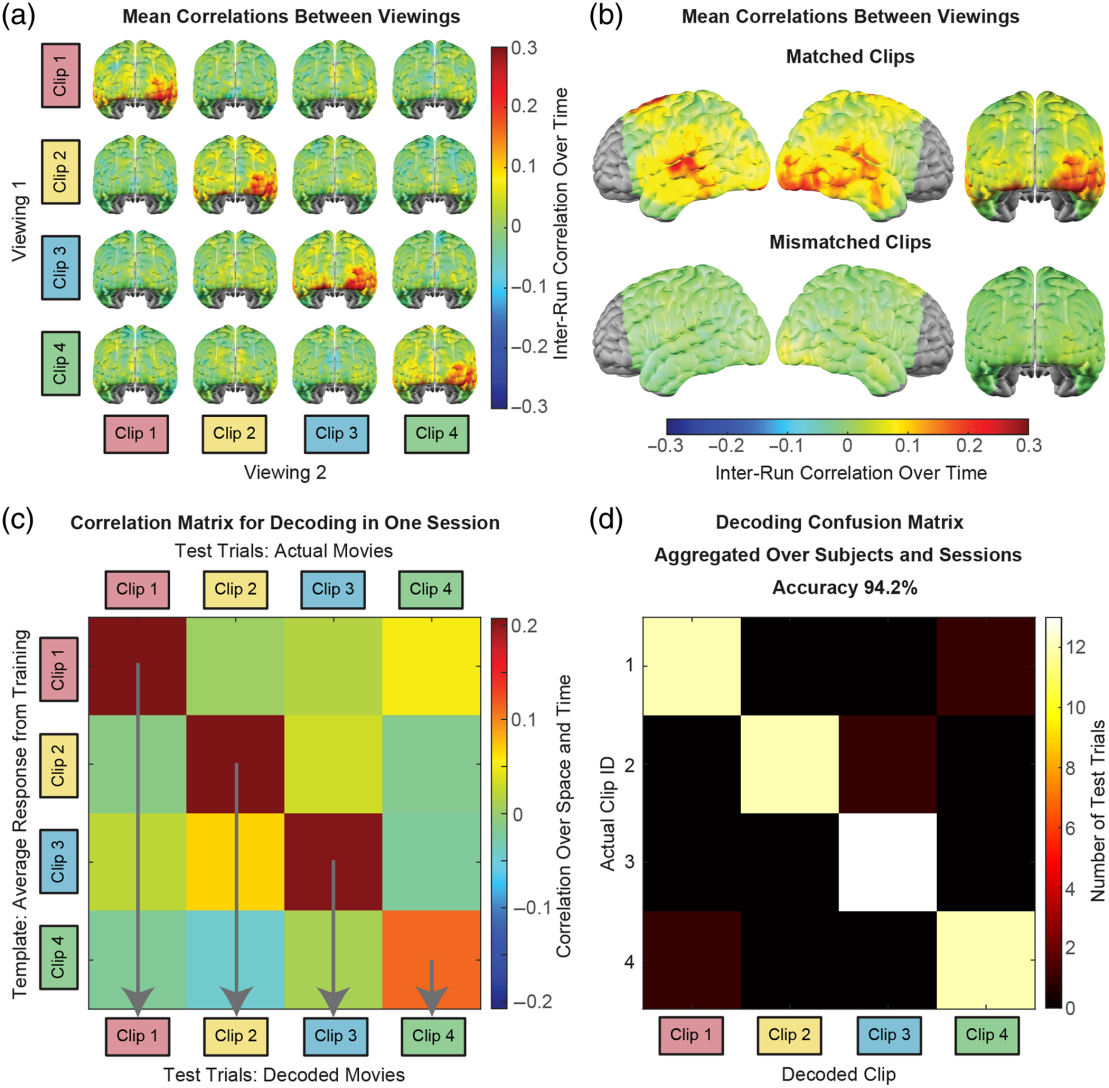
Decoding movie identity from HD-DOT data by template matching. (a) Comparing cortical responses imaged with HD-DOT between independent viewings of every possible pairing of movie clips presented in an imaging session reveals strong correlations between runs in which the participant was presented with the same movie clip. The maps presented here are averaged across all imaging sessions. (b) Mean inter-run correlation maps across all possible within-session pairings of matched and mismatched movie runs in the entire dataset (52 matched movie runs, 156 mismatched movie runs). (c) A template-matching strategy can hence be taken to decode which of a set of movies a participant was viewing from spatiotemporal correlations of their HD-DOT data. (d) Decoding results aggregated across the 13 imaging sessions. The bright main diagonal of the confusion matrix illustrates that the decoded clip usually matched the clip that was truly presented, with a calculated overall accuracy of 94.2±4.2% (mean ± SEM).

To examine which brain areas responded most reliably to each clip, we also computed Pearson correlation maps $c_{n,m}[v]$, in which the correlation at each voxel was computed only over time [e.g., Fig. 1(a)]:

$$T_{n,v}^{\prime }=[\begin{array}{c} \begin{array}{c} T_{n}[v,1]-\overset{¯}{T_{n}}[v]\\ \vdots \end{array}\\ T_{n}[v,t]-\overset{¯}{T_{n}}[v]\\ \begin{array}{c} \vdots \\ T_{n}[v,N_{T}]-\overset{¯}{T_{n}}[v] \end{array} \end{array}]\;S_{m,v}^{\prime }=[\begin{array}{c} \begin{array}{c} S_{m}[v,1]-\overset{¯}{S_{m}}[v]\\ \vdots \end{array}\\ S_{m}[v,t]-\overset{¯}{S_{m}}[v]\\ \begin{array}{c} \vdots \\ S_{m}[v,N_{T}]-\overset{¯}{S_{m}}[v] \end{array} \end{array}],$$ (4)

$$c_{n,m}[v]=\frac{T_{n,v}^{\prime }\cdot S_{m,v}^{\prime }}{\left|T_{n,v}^{\prime }||S_{m,v}^{\prime }\right|}.$$ (5)

Here, $\overset{¯}{T_{n}}[v]$ and $\overset{¯}{S_{m}}[v]$ denote the means of $T_{n}[v,t]$ and $S_{m}[v,t]$, respectively, over all time points in the window of interest W. These correlation maps were generated individually for each participant and session prior to averaging across all available datasets.

## 3 Results

### 3.1 Feasibility of Decoding Movie Viewing HD-DOT Data by Template Matching

Effective decoding of stimulus information from neuroimaging data depends on the measured brain responses being sensitive and specific to each stimulus condition and reproducible across instances of each condition. Voxel-wise correlations between oxyhemoglobin signal time courses were computed for every possible pairing of movie clips within each imaging session and then averaged across imaging sessions [Fig. 1(a)]. Strong positive correlations in the occipital and temporal cortex were consistently seen along the main diagonal of the correlation matrix but not in the off-diagonal maps, i.e., for independent runs in which participants were presented with the same movie clip both times but not for mismatched movie clips. This difference between matched and mismatched movie responses was further evident by averaging correlations across all within-session pairings of matched movie runs (52 run pairs) and mismatched movie runs (156 run pairs) across all imaging sessions (13 sessions each featuring four movie clips screened twice each) [Fig. 1(b)]. The reproducible, movie-specific responses mapped by HD-DOT indicated that the movie presented during a test run could be decoded from the participant’s HD-DOT data by comparison to template responses from training data collected during the same imaging session [Fig. 1(c)]. The performance of this template-matching approach to movie decoding from HD-DOT data was evaluated across 13 imaging sessions, involving three participants viewing four to five different sets of four unique movie clips per session [Fig. 1(d)]. The bright main diagonal of the confusion matrix reveals that the decoded clip most often matched the clip that was presented. Decoding accuracy was 94.2±4.2% [mean ± standard error of the mean (SEM)], significantly greater than the chance level of 25% ($p<10^{-6}$, binomial test), and reflected by a strong effect size, d=4.62. Main text figures present decoding based on oxyhemoglobin signals, while decoding performance metrics for the deoxyhemoglobin and total hemoglobin signals are included for comparison in Figs. S1 and S2 in the Supplementary Material.

### 3.2 Factors Affecting Decoding Performance

**Fig. 2:**
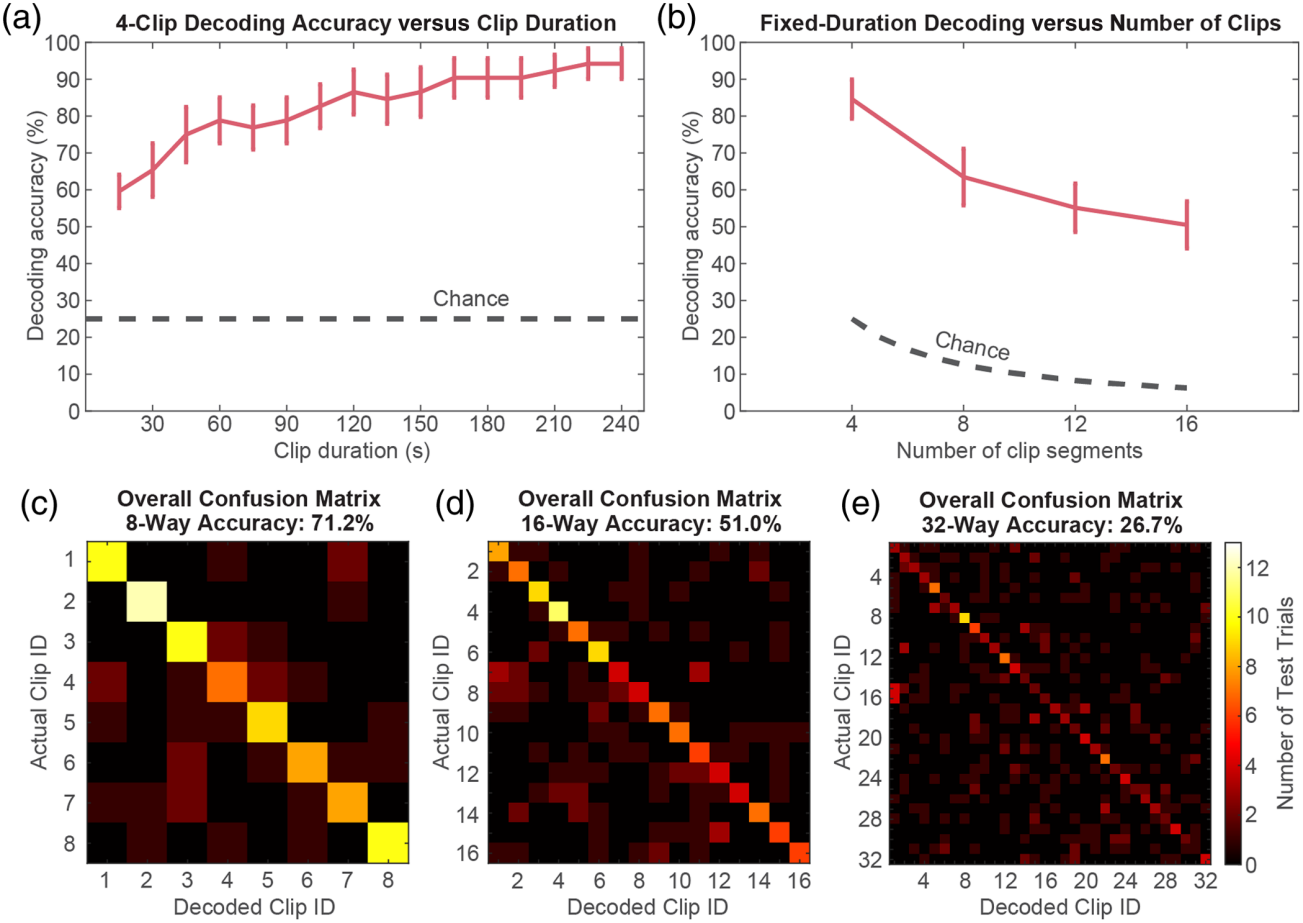
Factors affecting decoding performance. (a) Mean decoding accuracy across participants and sessions as a function of trial duration. Error bars denote SEM across participants and sessions. (b) Mean decoding accuracy as a function of the number of decoding targets. Error bars again denote SEM across participants and sessions. (c)–(e) Confusion matrices for decoding eight 105-s clip segments (c), sixteen 45-s clip segments (d), and thirty-two 15-s clip segments. Although accuracy (indicated by the brightness of the main diagonal) decreased as expected by making the decoding more challenging with an increasing number of targets and decreasing amounts of template and test data, mean accuracy remained significantly greater than chance in all these cases ($p<10^{-6}$, binomial test), with strong effect size (Cohen’s d>1.4), as quantified in Table 1.

Given the encouraging results for decoding clip identity using four trials of substantial duration, we systematically investigated the effects of trial durations and the number of decoding targets on decoding accuracy. First, the duration of each template and test trial was varied in 15-s decrements from 4 min down to 15 s in length to test decoding with smaller quantities of input data. Decoding accuracy was reevaluated across participants and sessions for each clip duration [Fig. 2(a)]. Mean decoding accuracy improved as expected with increasing trial duration but remained substantially above chance in all tested cases. Another common approach to challenging decoding algorithms is to increase the number of possible classification options, which creates a higher likelihood of incorrect classification, effectively stress-testing the decoder. To this end, each movie clip was split into four shorter segments, and decoding accuracy was reevaluated upon varying the total number of templates and decoding choices from four to 16 in increments of four while holding the trial duration constant at 45 s [Fig. 2(b)]. As anticipated, accuracy decreased with an increasing number of options, but performance remained well above chance even for the 16-way classification.

Both the trial duration and the number of templates were then varied in concert, and confusion matrices were computed for eight-way decoding with 105-s-long clips [Fig. 2(c)], 16-way decoding with 45-s-long clips [Fig. 2(d)], and 32-way decoding with 15-s-long clips [Fig. 2(e)]. These timings were chosen to still allow adequate intervals between decoding trials. More challenging decoding was associated with more decoding errors as expected, but accuracy remained significantly above chance with a strong effect size in all cases ($p<10^{-6}$, binomial test, Cohen’s d>1.4) (Table 1).

**Table 1:** Effect of varying both number of choices and trial length on movie decoding accuracy.

Number of choices	Trial length (s)	Chance decoding accuracy (%)	Observed decoding accuracy (mean ± SEM) (%)	p for significance above chance	Cohen’s d for effect size above chance
4	240	25.0	94.2 ± 4.2	$<10^{-6}$	4.62
8	105	12.5	71.2 ± 7.8	$<10^{-6}$	2.09
16	45	6.25	51.0 ± 6.5	$<10^{-6}$	1.90
32	15	3.13	26.7 ± 4.5	$<10^{-6}$	1.45

SEM denotes the standard error of the mean.

**Fig. 3:**
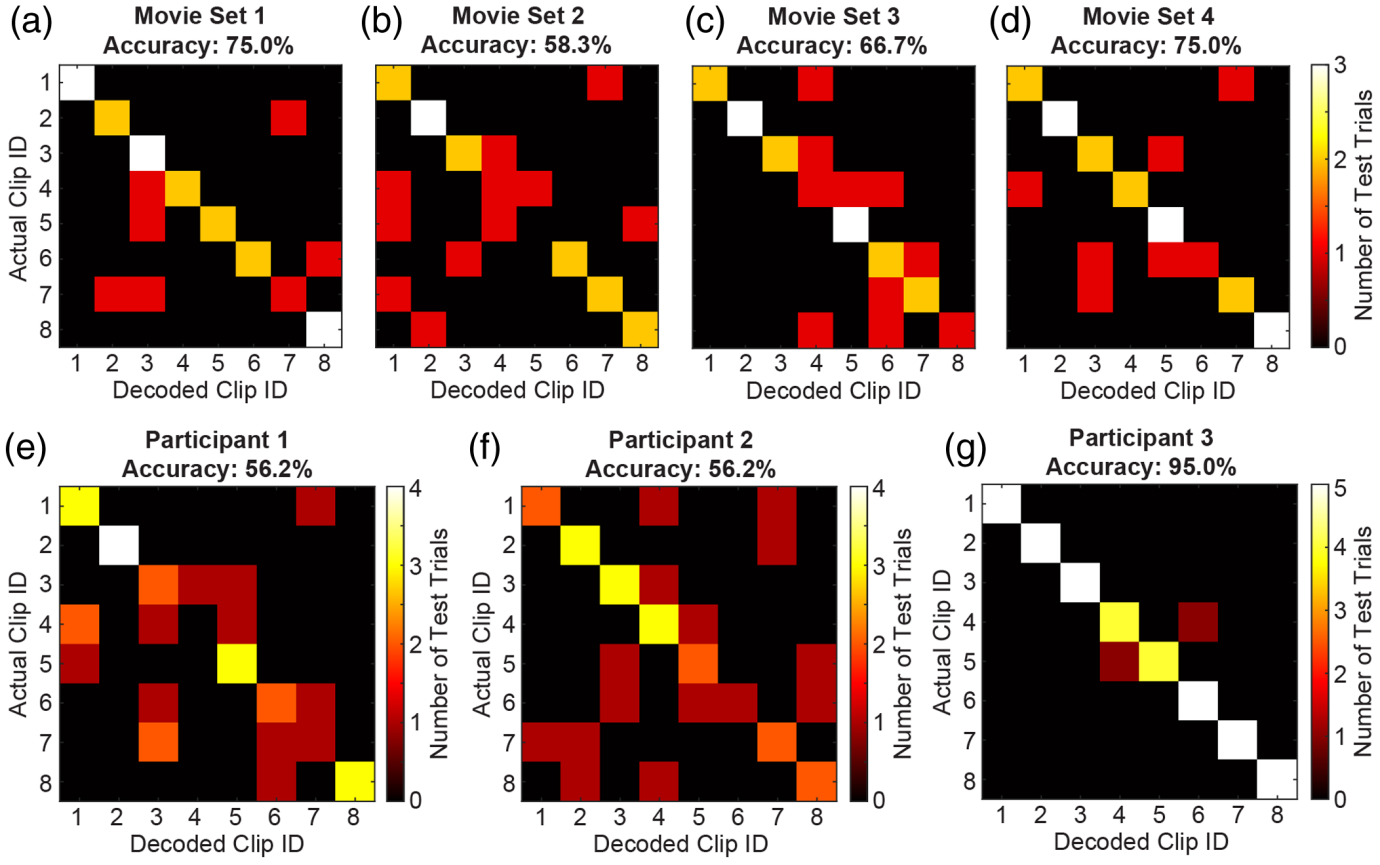
Eight-clip, 105-s decoding across different sets of movies and participants (wherein chance accuracy = 12.5%). (a)–(d) Confusion matrices across three participants for each of four different sets of movie clips. (e)–(g) Confusion matrices across imaging sessions for each of the three participants. Maximum values for each confusion matrix are dependent on the total number of test trials, which varied slightly between movie sets and participants based on data collection.

As the analysis thus far aggregated observations across multiple sets of movies and participants, we then explored variability between movies and between individuals, reasoning that associated differences in factors such as movie features, data quality, and participant engagement could influence decoding performance. Decoding performance was evaluated separately for each set of movies [Figs. 3(a)–3(d)] and each participant [Figs. 3(e)–3(g)]. Although the bright main diagonal indicated effective decoding in all cases, more errors (reflected by off-diagonal elements of the confusion matrices) were observed for some sessions and participants than others. Decoding accuracy was quantified separately in each case and noted to be consistently well above chance. The inter-run correlation was computed and mapped individually for each participant to assess the high correlation regions driving decoding performance (Fig. S3 in the Supplementary Material).

### 3.3 Decoding Within Auditory and Visual Regions of Interest

**Fig. 4:**
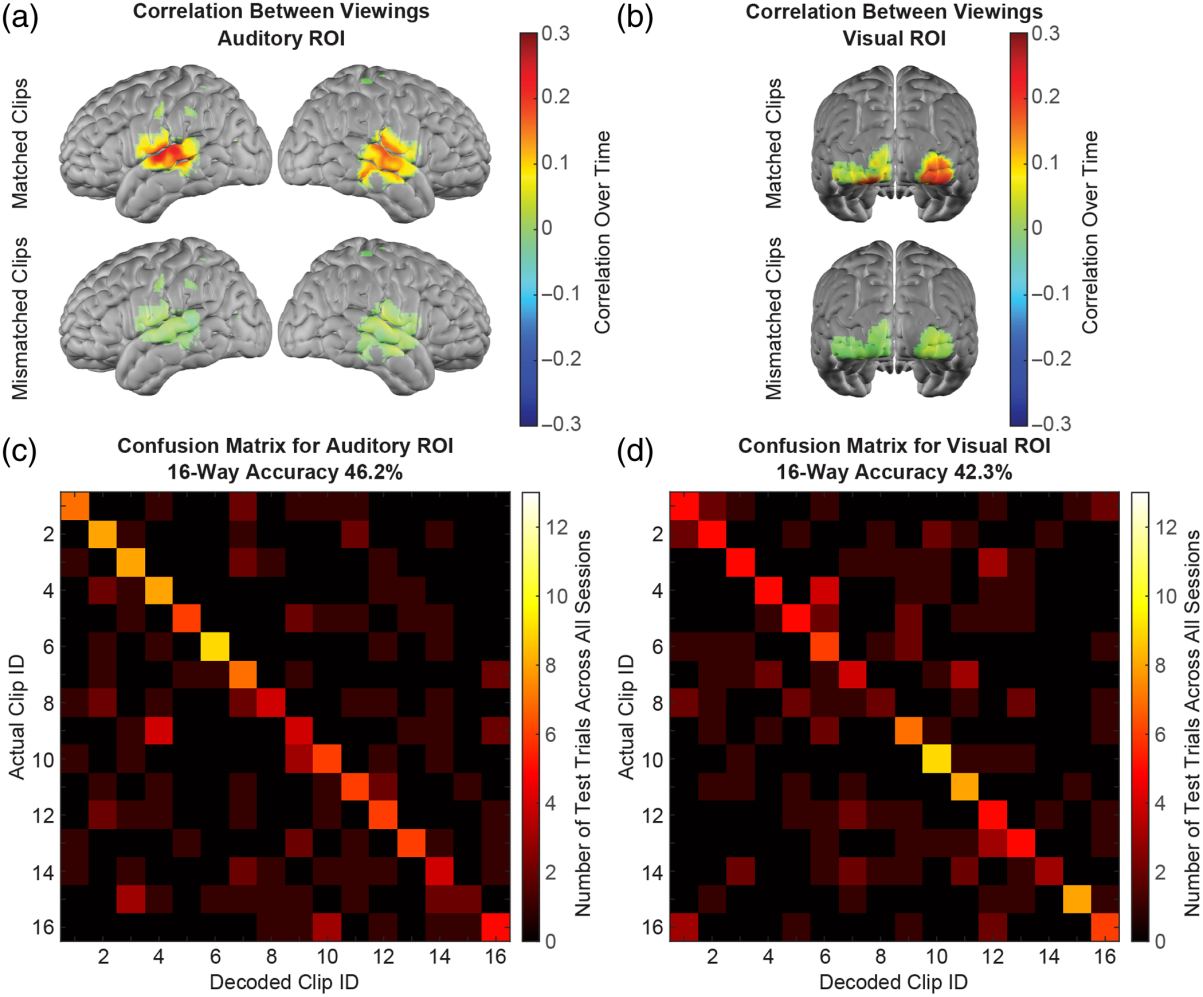
Decoding within auditory and visual ROIs. (a) Mapping inter-run correlations across all sessions shows high correlations between responses to matched and not mismatched clips within a pre-defined, task-based, auditory ROI. (b) Mapping inter-run correlations across all sessions shows high correlations between responses to matched and not mismatched clips within a pre-defined, task-based, visual ROI. (c) Confusion matrix illustrating that decoding using only data from within the auditory ROI is effective in the complex case with 16 choices and 45 s of data per template and trial (mean ± SEM accuracy = 46.2 ± 5.2%, whereas chance = 6.25%). (d) Confusion matrix showing that decoding using only data from within the visual ROI is also effective in the case with 16 choices and 45 s of data per template and trial (mean ± SEM accuracy = 42.3 ± 3.8%, whereas chance = 6.25%).

It was observed that signal correlations for matched movie viewing runs were strong in both the temporal cortex and occipital cortex [Fig. 1(b)], where responses to auditory stimuli and visual stimuli, respectively, are typically localized and have been previously mapped with HD-DOT.^26^^,^^35^ This observation inspired the evaluation of whether the presumed multisensory decoding was truly drawing on the cortical encoding of both sensory modalities and whether effective decoding of complex naturalistic stimuli might also be feasible based on either auditory or visual responses alone. The template matching analysis was repeated using template and test data confined to auditory and visual ROIs, defined based on block-design task data and neuroanatomy. Mapping correlations across matched movie runs and mismatched run pairs from every imaging session revealed strong positive correlations within both ROIs [Figs. 4(a) and 4(b)]. Template matching within each of these ROIs yielded decoding performance comparable to those obtained across the full field of view in both simpler and more complex versions of the experiment, with decoding accuracy significantly above chance ($p<10^{-6}$, binomial test) and with strong effect sizes (Cohen’s d>1.2) in all cases. The results are illustrated for the case of 16-way decoding with 45-s-long clip segments [Figs. 4(c) and 4(d)]. Preliminary decoding results using data from auditory-only and visual-only stimulus presentations similarly illustrated sustained decoding performance with 100% accuracy for these two imaging sessions (Fig. S4 in the Supplementary Material).

## 4 Discussion

In summary, we used an HD-DOT data set from neurotypical adults watching audiovisual movie clips to evaluate the feasibility and performance of decoding naturalistic auditory and visual stimuli from optical neuroimaging data. Based on the reproducibility and specificity of movie-evoked brain activity captured by HD-DOT imaging, we used a template matching strategy to first decode which of four movies participants had viewed from 4-min-long trials with 94.2±4.2% accuracy (Fig. 1). Decoding accuracy remained significantly above chance with robust effect sizes as trial duration was systematically reduced to 15 s [Fig. 2(a)] and as the number of template and trial types was increased up to 32 [Fig. 2(b)]. Mean decoding accuracy also remained above 85% for four-way decoding and above 40% for 16-way decoding when using template and test data confined to auditory or visual ROIs (Fig. 4). Decoding was similarly effective with purely auditory and purely visual stimuli. Altogether, we establish that naturalistic auditory and visual stimulus information encoded in cortical hemodynamics can be decoded effectively from HD-DOT measurements.

### 4.1 Toward More Complex Decoding with Human Optical Neuroimaging Data

The current study advances the complexity of decoding achieved with human optical neuroimaging data with regard to both the nature of the stimuli used and the number of possible targets effectively classified. Most fNIRS decoding studies have focused on classifying between two and six classification targets,^19^^,^^39^^,^^40^ although more recent work using high-density systems has begun to expand this range.^28^ Moreover, previous fNIRS and HD-DOT studies have demonstrated effective decoding of purely visual^30^^,^^33^ or purely auditory^19^^,^^24^ stimuli from optical neuroimaging data. FNIRS research beginning to explore multisensory stimulus decoding has achieved the binary classification of less naturalistic auditory and visual stimulus combinations.^19^ Furthermore, previous research has shown that naturalistic viewing of movie stimuli evokes reproducible patterns of distributed brain activity that can be reliably captured using HD-DOT, suggesting the feasibility of effectively decoding such stimuli.^33^^–^^35^ The current study extends this prior body of work by establishing effective decoding of naturalistic audiovisual movie stimulus identity with four to 32 decoding targets. Although the number of participants is low in the current study, the amount of data collected is large with a minimum of 175 min of movie viewing data collected per participant. This approach of precision data collection is common in naturalistic decoding studies, with many influential decoding studies to date focused on three to five highly sampled participants.^1^^,^^3^^,^^6^^,^^33^ In addition, the high channel count of our HD-DOT imaging system, with ∼2000 viable measurement channels, provides significantly larger quantities of data than most traditional fNIRS studies, which typically report channel counts of less than 100 measurements. Future work could involve collecting additional datasets on more participants or expanding this study to additional populations such as children for whom these animated movie clips would be well-suited stimuli; however, these ideas lie beyond the scope of the current study.

The classification accuracy observed using single runs to construct templates for decoding single trials indicates both the degree of replicability of responses to a given movie from run to run and the high discernibility of responses between individual viewings of different movies (Fig. 1). The performance baseline established using relatively generous parameters of 4-min long trials with four classification options supported challenging the template matching algorithm and HD-DOT data through manipulations such as decreasing trial duration and increasing the number of trial types. Mean decoding accuracy decreased as anticipated on reducing trial duration but remained significantly above chance even with just 15 s of data [Fig. 2(a)]. Likewise, classification errors became more common as the number of templates and trial types increased, but accuracy remained better than chance in all cases tested [Fig. 2(b)]. Significance testing and effect sizes illustrated robust decoding performance for all combinations of tested parameters, including as many as 32 trial types with 15-s trial durations [Figs. 2(c)–2(e) and Table 1]. Differences in raw data quality and participant engagement were considered possible contributors to observed variance in decoding performance between participants and movies (Fig. 3). This variation in performance is comparable to that observed between participants in prior studies of visual decoding.^30^^,^^33^

The trial durations used in this study are admittedly longer than those used in prior HD-DOT decoding research classifying checkerboard stimulus location from single second frames for instance.^30^ However, the stimuli used in the current study are also significantly more dynamic, multi-dimensional, and variable than visual checkerboards. FMRI studies have accomplished framewise decoding of naturalistic stimuli utilizing large quantities of training data amassed across imaging sessions to support more complex decoding algorithms.^6^ The current work makes an important first step in this direction and represents an important advance for the optical neuroimaging field.

### 4.2 Decoding Isolated Auditory and Visual Responses

Decoding cortical responses to visual scenes and auditory content including naturalistic speech represent important steps toward clinically relevant applications such as eventually decoding imagined scenes and intended speech production. Moreover, movie stimuli include semantic content embedded in the narrative of the clips that may be encoded in widely distributed regions of the cortex, such that movie decoding may also represent a first step toward semantic decoding.^1^^–^^3^^,^^6^^,^^11^^,^^51^^–^^53^ Effective audiovisual movie decoding thus warranted further investigation of the stimulus components potentially contributing to decoding. Given the strong inter-run correlations observed in both temporal areas associated with auditory processing and occipital areas associated with visual processing, the ROI-confined analysis attempted to disentangle the decoding of auditory and visual responses. These ROIs included regions of the auditory and visual cortex derived from isolated functional localizer tasks. Decoding performance decreased slightly but remained robust (based on both significance testing and effect sizes) with template matching confined to either ROI, indicating that multisensory decoding was not solely driven by either sensory modality on its own but rather drew on the combined cortical encoding of both auditory and visual stimulus features. The increased decoding performance when considering the entire HD-DOT field of view indicates the added value from wide-field or whole-head coverage, potentially a result of capturing additional signals contained in semantic or other higher-order cortical regions beyond early sensory processing areas.^1^^,^^2^ Furthermore, this result indicated the scope for decoding naturalistic auditory or visual information independent of one another using HD-DOT. Granted, neither ROI definitively excludes responses to the other sensory modality; for instance, responses to visual features such as faces have been reported in the right superior temporal sulcus,^54^ which partially overlaps with our task-data-derived auditory ROI due to proximity to the superior temporal gyrus where primary auditory responses are centered. The purely auditory and purely visual stimulus data thus allowed for a more controlled preliminary evaluation of auditory and visual decoding in isolation. Decoding remained accurate across these sessions as well, for both audio clips presented sans visuals and silent movies stripped of audio. The promising results of this pilot analysis support further studies focused on decoding both sensory modalities with HD-DOT. Auditory decoding in particular had yet to be explored with HD-DOT data, and the current study presents a critical first step in this endeavor. Further research using a broader range of stimuli and nuanced stimulus encoding models can evaluate how much the presented decoding is driven by different stimulus features ranging from low-level auditory and visual characteristics to high-level semantic content.

### 4.3 Future Directions

Previous studies using other imaging modalities have accomplished impressively detailed and accurate decoding of visual, auditory, and other information from recordings of brain activity. Of note, fMRI recordings of cortex activity have been used to reconstruct naturalistic images, dynamic silent visual scenes, and semantic content from movies and podcasts.^1^^,^^3^^,^^6^^,^^9^^,^^10^ In addition, electrocorticographic recordings from brain areas associated with language and motor function have been used to synthesize intelligible speech in epilepsy patients.^4^^,^^12^ A common thread amongst this research is the usage of data sets containing only a few highly sampled participants in lieu of larger sample sizes. This data set structure is often necessary to accrue sufficient training and testing data for the desired decoding task. Our template-matching classification task required a minimum of two repetitions of the movie clips during one imaging session. More complex, model-based decoding algorithms would hinge on larger sets of optical neuroimaging data collected across multiple sessions.^1^^,^^3^^,^^6^ Previous fMRI studies of visual decoding have used receptive field models to decode novel static images,^9^ motion energy encoding models to reconstruct dynamic visual stimuli,^3^ and convolutional neural networks to encode and decode hierarchical layers of visual stimulus features and neural responses.^7^^,^^10^ Other fMRI studies have used a variety of methods, including multivariate pattern analysis, ridge regression, and generative models, to encode and decode acoustic, articulatory, and semantic content of speech^1^^,^^55^^–^^59^ as well as other auditory stimuli such as music.^60^ In addition, ECoG studies have taken various approaches, from linear classification methods to a variety of feature encoding models, to decode articulatory or spectral features during speech production and thereafter synthesize intelligible speech from electrical recordings of brain activity.^4^^,^^12^^,^^61^ More recently, highly accurate and efficient brain-to-text communication has also been achieved in a quadriplegic patient by decoding motor cortex activity sampled with surgically implanted intracortical microelectrodes during imagined handwriting.^13^ However, these studies are currently constrained in their end-applications by the logistical limitations of fMRI and the invasiveness of ECoG. Although considered beyond the focus of the current study, future work could also capitalize on the greater sampling rate feasible with optical recording methods compared with MRI to explore whether additional information can be extracted and decoded from data sampled at higher frequencies. Additional feature selection techniques could also be implemented, including further filtering the data for noisy measurements, restricting the spatial information to the most relevant voxels, or using other dimensionality reduction techniques to isolate relevant measures. Although the current study was limited to simpler decoding methods, the results presented show that HD-DOT recordings of brain activity have high enough information content and repeatability for decoding complex stimuli. This finding motivates further studies applying more elaborate decoding algorithms to HD-DOT data, potentially extending prior groundbreaking fMRI and ECoG studies toward more widespread translation in real-world contexts.

In addition to investigating more elaborate decoding and developing clinical applications, future studies could also push toward decoding during increasingly natural paradigms. While free viewing of audiovisual movies provides a segue from block-design tasks, imaging studies have begun to explore even more naturalistic paradigms such as immersive three-dimensional movies, interactive virtual reality, and face-to-face human interactions.^62^^,^^63^ Although harder to control, these interactive paradigms attempt to enhance the ecological validity of laboratory research and move closer to real-world applications. With recent advancements in fiberless, wearable HD-DOT technology, this imaging modality will prove increasingly well suited as a tool for mapping and decoding information during natural paradigms.^64^

Altogether, the current study establishes the feasibility of decoding complex audiovisual stimuli from optical neuroimaging data without the logistical constraints of alternative neural recording methods. Furthermore, the study extends prior work advancing and characterizing decoding performance with optical neuroimaging and illustrates the associated fidelity of HD-DOT signals. These findings comprise major steps forward for decoding with optical neuroimaging and encourage future studies geared toward more elaborate decoding, more natural paradigms, and clinical applications.

## Supplementary Material



## Data Availability

The data and code presented in this article are publicly available at https://www.nitrc.org/projects/neurodot.
