# Supplementary material for: Multisensory naturalistic decoding with high-density diffuse optical tomography
Source: Neurophotonics. 2025 Jan 23;12(1):015002. doi: 10.1117/1.NPh.12.1.015002 (PMC11755382; doi:10.1117/1.NPh.12.1.015002)
Supplement: Supplementary file 1 [file NPh_012_015002_SD001.pdf]

Supplemental Table ST1: Data Collection in Adults.

Subject	Number of Auditory Localizer Runs	Number of Visual Localizer Runs	Number of Movie Viewing Runs	Total Number of Movie Viewing Minutes
A01	2	1	32	175.6
A02	2	2	32	175.6
A04	1	2	40	216.9
Total	5	5	104	568.1

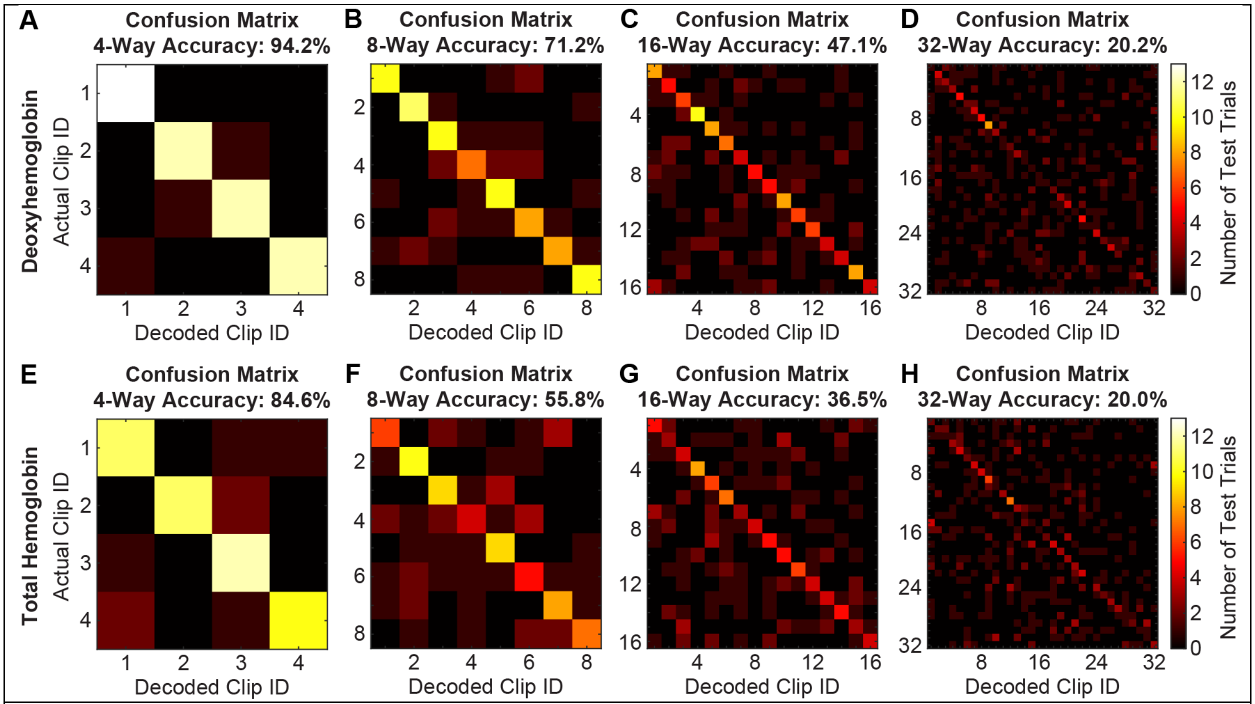

Supplemental Figure 1: Confusion matrices for decoding analyses repeated with deoxyhemoglobin and total hemoglobin signals in lieu of oxyhemoglobin reveal comparable results across contrasts.

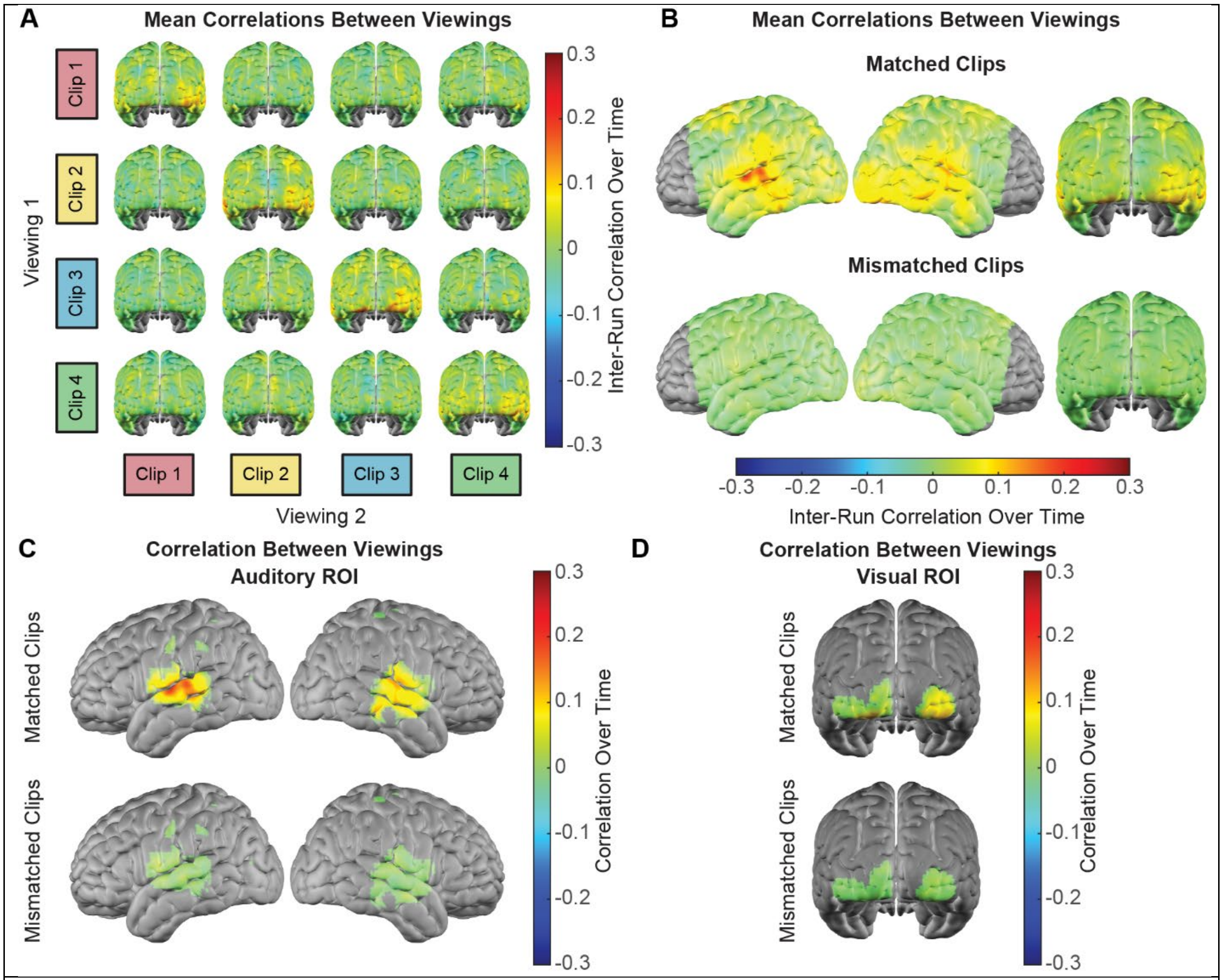

Supplemental Figure 2: Deoxyhemoglobin cortical maps for movie correlations. (A) Averaging the correlations across all imaging sessions for each session's movie clips results in higher correlations for matched (on diagonal) versus mismatched (off diagonal) movie clips. Mean inter-run correlation maps were computed across all possible within-session pairings of matched and mismatched movie clips using deoxyhemoglobin signals across the entire field of view (B), and within auditory (C) and visual (D) regions of interest.

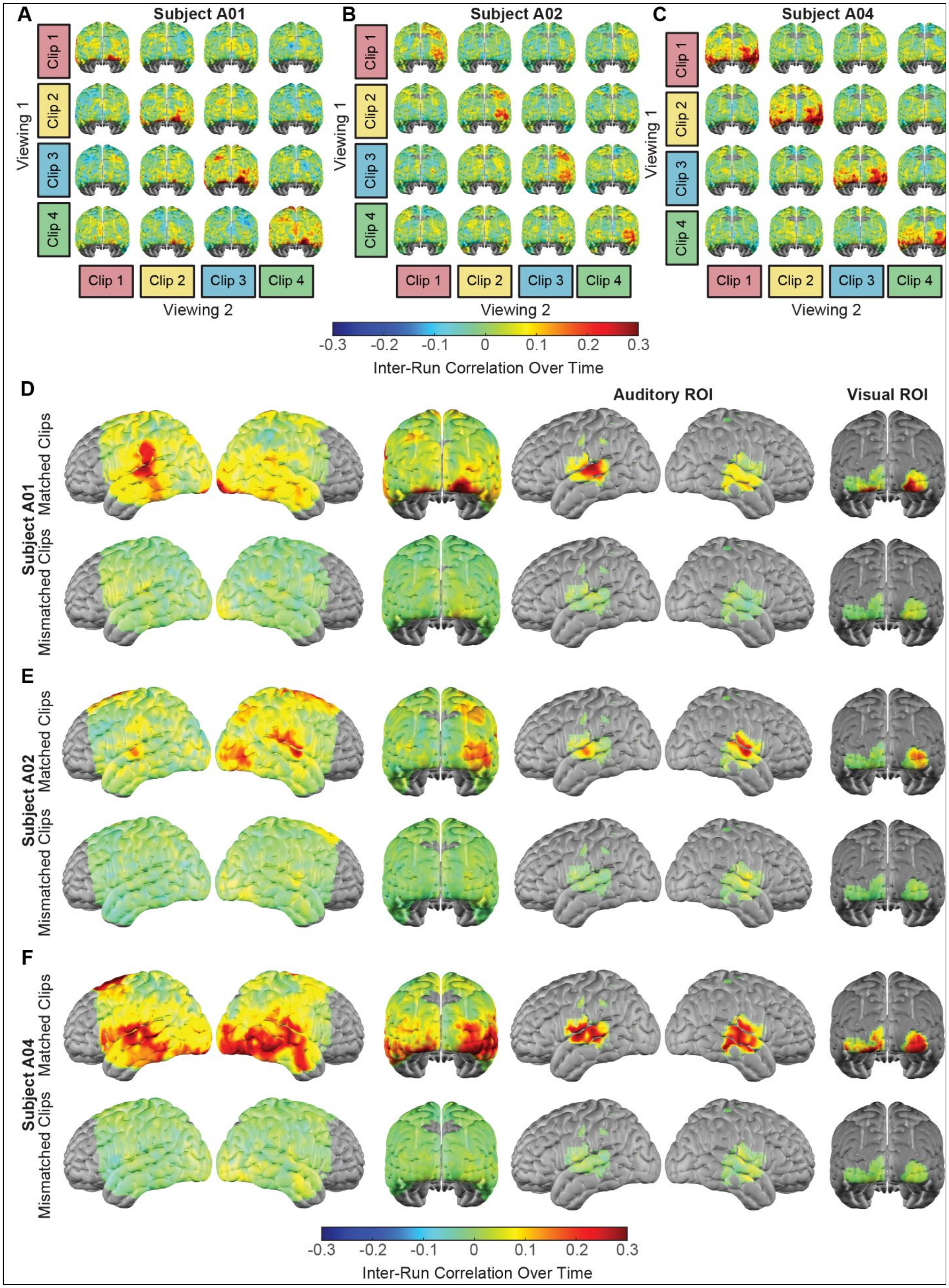
A
Subject A01
B
Subject A02
C
Subject A04
Viewing 1
Clip 1
Clip 2
Clip 3
Clip 4
Viewing 2
-0.3 -0.2 -0.1 0 0.1 0.2 0.3
Inter-Run Correlation Over Time
D
Auditory ROI
Visual ROI
Subject A01
Matched Clips
Mismatched Clips
E
Subject A02
Matched Clips
Mismatched Clips
F
Subject A04
Matched Clips
Mismatched Clips
-0.3 -0.2 -0.1 0 0.1 0.2 0.3
Inter-Run Correlation Over Time

Supplemental Figure 3: Oxyhemoglobin cortical mapping across individual participants. An average correlation matrix was computed across each session within an individual participant to evaluate the matched (on diagonal) and mismatched (off diagonal) movie clip correlations for subjects A01 (A), A02 (B), and A04 (C). The mean inter-run correlation was computed across all matched and mismatched movie clip pairs and plotted across the entire field of view and within auditory and visual regions of interest for subjects A01 (D), A02 (E), and A04 (F).

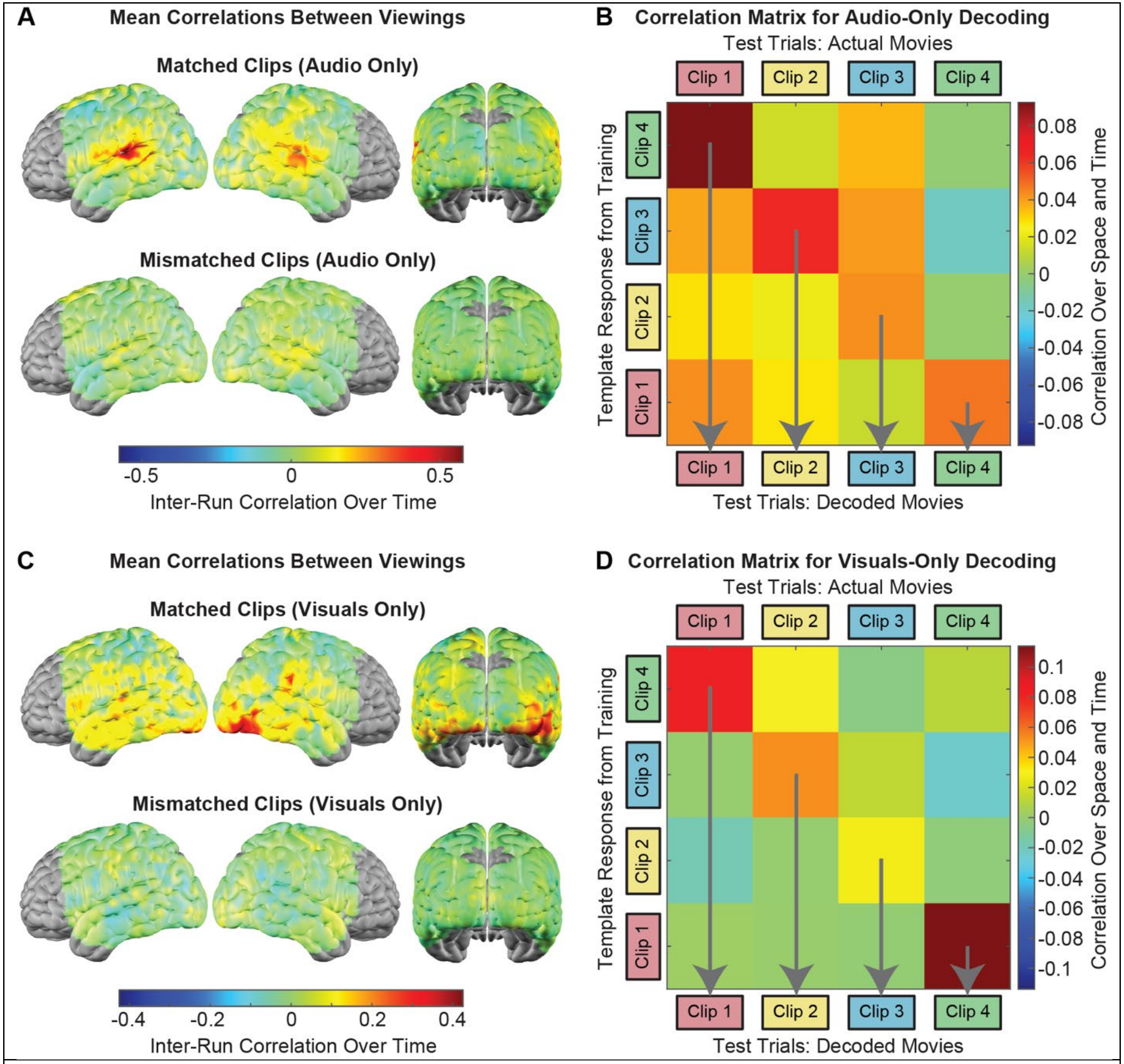

Supplemental Figure 4: Decoding purely auditory and purely visual naturalistic stimuli from HD-DOT data. (A) Participant A04 was presented with four different audio clips without any visuals twice each over the course of an imaging session. Comparing cortical responses imaged with HD-DOT between independent presentations of every possible pairing of audio clips reveals strong correlations in the superior temporal gyrus for runs in which the participant was

presented with matched clips but not mismatched clips. (B) Decoding of audio clip identity by spatiotemporal template matching. (C) Participant A04 was presented with four different silent movie clips twice each over the course of an imaging session. Comparing cortical responses imaged with HD-DOT between independent presentations of every possible pairing of clips reveals strong correlations in occipital cortex between runs in which the participant was presented with the same clip but not mismatched clips. (D) Decoding of silent movie identity by spatiotemporal template matching.
